# Two Loci, *RiAF3* and *RiAF4*, Contribute to the Annual-Fruiting Trait in *Rubus*

**DOI:** 10.3389/fpls.2019.01341

**Published:** 2019-10-25

**Authors:** Rubina Jibran, Jessica Spencer, Gina Fernandez, Amparo Monfort, Mourad Mnejja, Helge Dzierzon, Jibran Tahir, Kevin Davies, David Chagné, Toshi M. Foster

**Affiliations:** ^1^The New Zealand Institute for Plant & Food Research Limited, Palmerston North Research Centre, Palmerston North, New Zealand; ^2^Department of Horticultural Science, North Carolina State University, Raleigh, NC, United States; ^3^IRTA (Institut de Recerca I Tecnologia Agroalimentàries), Barcelona, Spain; ^4^Centre for Research in Agricultural Genomics (CRAG) CSIC-IRTA-UAB-UB, Barcelona, Spain

**Keywords:** marker-assisted selection, primocane, floricane, comparative mapping, RNA sequencing, annual-fruiting, biennial-fruiting

## Abstract

Most *Rubus* species have a biennial cycle of flowering and fruiting with an intervening period of winter dormancy, in common with many perennial fruit crops. Annual-fruiting (AF) varieties of raspberry (*Rubus idaeus* and *Rubus occidentalis* L.) and blackberry (*Rubus* subgenus *Rubus*) are able to flower and fruit in one growing season, without the intervening dormant period normally required in biennial-fruiting (BF) varieties. We used a red raspberry (*R. idaeus*) population segregating for AF obtained from a cross between NC493 and ‘Chilliwack’ to identify genetic factors controlling AF. Genotyping by sequencing (GBS) was used to generate saturated linkage maps in both parents. Trait mapping in this population indicated that AF is controlled by two newly identified loci (*RiAF3* and *RiAF4*) located on *Rubus* linkage groups (LGs) 3 and 4. The location of these loci was analyzed using single-nucleotide polymorphism (SNP) markers on independent red raspberry and blackberry populations segregating for the AF trait. This confirmed that AF in *Rubus* is regulated by loci on LG 3 and 4, in addition to a previously reported locus on LG 7. Comparative RNAseq analysis at the time of floral bud differentiation in an AF and a BF variety revealed candidate genes potentially regulating the trait.

## Introduction

The *Rosoideae* subfamily of Rosaceae contains many economically important soft berry crops, including red and black raspberry (*Rubus idaeus* and *Rubus occidentalis* L. respectively), blackberry (*Rubus* subgenus *Rubus*) and strawberry (*Fragaria* species), which are renowned for their taste and health properties (Potter et al., 2007; Hummer and Janick, 2009; Shi et al., 2013; Simpson, 2018). Raspberry (*Rubus* sp.) is a shrub that initiates shoots (canes) from a perennial root system (Keep, 1988; Carew et al., 2000; Sønsteby and Heide, 2009; Heide et al., 2013; Graham and Simpson, 2018). Biennial-fruiting (BF) raspberry varieties (also called floricane-fruiting or summer-fruiting) initiate axillary floral buds toward autumn of the first year of growth, but these do not develop into fruit until spring/summer of the following year. Annual-fruiting (AF) varieties (also called primocane-fruiting or autumn-fruiting) initiate flowers in late spring/early summer that develop into fruit from summer until late autumn of the same year. In both AF and BF varieties, flowering and fruiting initiate from the shoot tip and develop basipetally after vegetative growth has stopped. The key developmental difference between the two flowering phenologies is that AF floral buds are initiated earlier and progress directly to fruit set, whereas floral initiation is normally followed by dormancy in BF types (Keep, 1988; Carew et al., 2000; Sønsteby and Heide, 2009; Heide et al., 2013).

Flowering time is controlled by complex interactions among endogenous factors, such as developmental pathways and hormones, as well as environmental cues, such as temperature and day length (Simpson and Dean, 2002; Song et al., 2018; Tabas-Madrid et al., 2018; Kinmonth-Schultz et al., 2019; Kozlov et al., 2019). A number of genes that integrate specific signals and either repress or activate flowering have been identified and characterized in model species, such as *Arabidopsis* and *Antirrhinum* (Simpson and Dean, 2002; Wigge et al., 2005; Khan et al., 2014; Blumel et al., 2015; Sasaki et al., 2015). Among these, CONSTANS (CO), FLOWERING LOCUS T (FT), SUPPRESSOR OF OVEREXPRESSION OF CONSTANS 1 (SOC1), FLOWERING LOCUS C (FLC), AGAMOUS1 (AG1), and LEAFY (LFY) are the best characterized flowering integrators (Yanofsky et al., 1990; Blazquez et al., 1997; Kardailsky et al., 1999; Kobayashi et al., 1999; Michaels and Amasino, 1999; Yoo et al., 2007; Deng et al., 2011; Pin and Nilsson, 2012; Song et al., 2018; Tabas-Madrid et al., 2018). For example, CO activates flowering under long days in both FLC-dependent and independent manners by activating flower promoters *FT* and *SOC1* (Kim et al., 2008; Michaels and Amasino, 2001). FLC regulates floral transition by repressing the key genes of flowering pathway, for example, *FT* and *SOC1* (Simpson and Dean, 2002; Crevillen and Dean, 2011). FLC antagonizes the flowering pathway in a dose-dependent manner, with FLC abundance being regulated by an interplay between epigenetic factors and RNA-processing factors, such as polyadenylation and splicing (Simpson, 2004). Recently, it was shown that CO accelerates flowering under long days but represses flowering under very short days (3 hours light) by regulating *FT* expression (Luccioni et al., 2019).

Although there is little information on the genes controlling flowering in raspberry, more is known about the environmental cues that stimulate flowering. For example, floral induction in BF varieties is triggered by a combination of decreased temperatures and shorter photoperiod (Fejer and Spangelo, 1974; Dale and Daubeny, 1987; Carew et al., 2000; Sønsteby and Heide, 2009; Hodnefjell et al., 2018). Although there is no absolute requirement for AF varieties to experience chilling in the prior season to initiate flowering, as newly initiated canes can progress through fruiting in a single season, the expression of AF in terms of floral consistency across canes and the total number of flowers is strongly influenced by chilling (Sønsteby and Heide, 2009).

Modern AF varieties of red and black raspberry have complex pedigrees because of interspecific hybridization with other *Rubus* species during their development, including *R.* *arcticus*, *R. odoratus*, and *R. spectabilis* (Keep, 1988; Lewers et al., 2005; Dossett et al., 2012). Many studies have been conducted to study the genetic inheritance of AF in raspberry and blackberry, along with an analogous continuous flowering trait in strawberry. Continuous or perpetual flowering in commercial strawberry (*Fragaria* × *ananassa*) is controlled by quantitative trait loci (QTLs) on linkage groups (LGs) 3, 4, and 7 (Gaston et al., 2013; Perrotte et al., 2016; Hackett et al., 2018), whereas AF in blackberry and red raspberry was suggested to be controlled by a recessive monogenic trait (Lewis, 1939; Haskell, 1960; Lopez-Medina et al., 2000). Castro et al. (2013) reported that in auto-tetraploid blackberry, this recessive locus was located on LG7.

Lewis (1939) demonstrated that the AF trait is controlled by a single recessive locus, later named “af” (Haskell, 1960). However, trait segregation analysis performed on various AF populations suggested an alternative possibility of multiple loci having minor effects on the expression of AF (Slate, 1940; Waldo and Darrow, 1941; Oberle and Moore, 1952; Ourecky, 1976; Fejer, 1977; Barrientos and Rodriguez, 1980). For example, Barrientos and Rodriguez (1980) suggested the possibility of partial dominance for the AF cultivar ‘Malling Exploit.’ Similarly, Fejer (1977) reported that inheritance of AF in mapping populations raised from a series of diallelic crosses among seven red raspberry cultivars was predominately additive and proposed that the genetic control for the trait could not be recessive. Thus, the genetic regulation of AF in raspberry is still unclear.

To address this issue, we constructed saturated linkage maps for the AF accession NC493 and the BF cultivar ‘Chilliwack’ to map genetic loci associated with control of AF. Trait mapping in this population indicated that AF is controlled by two newly identified loci (*RiAF3* and *RiAF4*) located on *Rubus* LGs 3 and 4. The location of these loci was verified in independent red raspberry and blackberry populations segregating for AF. In addition, we compared the transcriptomes of AF and BF axillary buds to identify candidate genes involved with the transcriptional regulation of the AF trait.

## Materials And Methods

### Plant Material and Assessment of Fruiting Phenotype

A segregating population of 131 F_1_ individuals was developed from a controlled cross made in 2004 between AF accession NC493 (*R. parvifolius* x *R. idaeus* ‘Cherokee’) and BF ‘Chilliwack’ (CW) (*R. idaeus*). The seedling population was planted in 2006 at the Sandhills Research Station, Jackson Springs, NC, USA. In 2008, the 131 NC493 x CW progeny were assessed biweekly from July to September for the presence or absence of AF by determining whether flowers or fruits were present on the primocanes (canes initiated that season). The population was again evaluated in 2009 for AF on a weekly to biweekly basis from June to August, except for two individuals that died over the winter.

Three families of red raspberry (*R. idaeus*), x16.093, x16.109, and x16.111 of 47, 55, and 49 individuals, respectively, were developed from controlled crosses between AF and BF parents within the Plant & Food Research (PFR) breeding program and planted in 2017 at the PFR site located at Motueka, New Zealand. These populations were phenotyped for the presence of AF in 2018.

A tetraploid blackberry (*R.* subgenus *Rubus*) mapping population (C1) was generated from a cross between BF RM44 and AF RM63 from the IRTA-PLANASA breeding program. The population was planted in 2015 at Cartaya, Spain. The parents and progeny were phenotyped for the presence of AF in 2016 and 2017.

### Genotyping by Sequencing

High molecular weight DNA was extracted from 100 mg of leaf tissue from each individual in the 131 progeny in the NC493 x CW family using a standard CTAB protocol (Doyle and Doyle, 1987). The genotyping by sequencing (GBS) method of Elshire et al. (2011) was used to obtain reduced representation of the genomes for the two parents and progeny of 83 individuals. The GBS library preparation protocol was first optimized for the red raspberry genome by digesting DNA from a few individuals with *Ape*K1, as described by Elshire et al. (2011). GBS libraries were then constructed for 83 individuals and the two parents. The libraries from 83 individuals were combined to make the final pooled library. The quantity and quality checks of the individual libraries and the pooled library were performed using a Qubit Fluorometer and a Fragment analyser, respectively. The pooled DNA library was dried and sent to the Australian Genome Research Facility for sequencing on two lanes of the Illumina HiSeq2500 platform using single-end sequencing chemistry.

The sequencing reads were demultiplexed based on GBS library preparation bar codes using the ea-utils.1.1.2-537 package (Richardson, 2013), and those reads starting with the approved bar code immediately followed by the remnant of the *Ape*K1 cleavage site sequence were retained for further analysis. The bar coded reads meeting the initial read quality criteria were aligned to the *R. occidentalis* genome assembly of ORUS 4115-3 v3.0 (Vanburen et al., 2018) (https://www.rosaceae.org/analysis/268) using Burrows-Wheeler Aligner (bwa/0.7.17) (Li and Durbin, 2009). Single-nucleotide polymorphism (SNP) calling and GBS data filtering were performed using the GATK pipeline (gatk/3.8.0) (McKenna et al., 2010) using default parameters. The GBS pipeline used to create a set of markers is available on Github at https://jupyterhub.powerplant.pfr.co.nz/user/cfprxj/notebooks/cfprxj/bioinf_Braspberry_GBS/Variant_calls_Braspberry_GATK_pipeline.ipynb. GATK_GBS analysis yielded a total of 284,146 SNPs between the two parents.

### Preparation of GBS Markers for Linkage Analysis

The SNP data were filtered and formatted for appropriate genetic segregation codes using MS Excel (Microsoft Corporation, USA). Markers segregating abxaa, aaxab, and abxab were selected for each parent using GATK and MS Excel. Homozygous SNP calls, such as A/A, G/G, T/T, and C/C, were converted into *aa* marker type, whereas heterozygous SNP calls (such as A/G, A/T, C/T, etc.) were converted into the *ab* marker type. Joinmap v5.0^®^ (Van Ooijen and Voorrips, 2001) was used to develop genetic linkage maps for each parent of the NC493 x CW population. A LOD score >6 was employed for grouping. Due to the high number of markers on each linkage group, the markers were then filtered based on chi-square values ranging from 0.1 to 7.0, and these selected loci were subsequently used to reconstruct the maps using regression mapping (Kosambi mapping function).

### Trait Mapping

Trait mapping was initially performed by including the AF phenotypes of both years in the GBS data set using Joinmap v5.0^®^ (Van Ooijen and Voorrips, 2001). As the phenotypes in the CW x NC493 population were scored as a presence-absence of AF, which is not quantitative and cannot be used for QTL mapping with methods such as interval mapping, a chi-square test was performed on all GBS markers that were heterozygous in only one parent to identify markers linked to AF. Chi-square values for the significant differences between the expected allelic frequencies and the observed allelic frequencies were calculated with the formula:

$${Χ}^{2}=\Sigma^{k}\text{i }=1\text{ [}{\left({\text{observed value}}_{\text{i}-}{\text{expected value}}_{\text{i}}\right)}^{2}/{\text{expected value}}_{\text{i}}\text{]}$$

GBS markers with chi-square test values between 5 and 20 and *p* values <0.05 were selected for identification of QTLs controlling to the trait. This filtering criterion yielded 26,925 abxaa markers that are heterozygous for the NC493 parent and 6,571 aaxab markers that are heterozygous for the CW parent. The markers that are heterozygous for one parent and homozygous for other parent were used for QTL mapping.

Further mapping was performed using abxaa markers (heterozygous for AF parent) located around the AF loci on the NC493 parental map. The phase of these markers was calculated using Joinmap v5.0^®^ (Van Ooijen and Voorrips, 2001), and they were ordered according to their physical location on the ORUS 4115-3 v3.0 *R. occidentalis* genome assembly (Vanburen et al., 2018). Bins of 10 to 12 markers within focal points spanning no more than 100 kb physical intervals were manually inspected. Focal points were evenly spaced throughout the region flanking the *RiAF4* locus at 0.1, 2, 3.1, 3.6, 3.8, 4.1, 4.5, 6, and 8 megabase pairs (Mb). Likely genotypic errors due to allelic dropout, a common feature in GBS data for heterozygous species and detectable as a single change in phase within a linked focal point, were manually corrected. A consensus genotype was then imputed for each focal point and compared to the neighboring focal points. The linkage between each focal point and the AF phenotype was examined to delimitate the most likely genomic interval flanking the AF loci.

### High-Resolution Melting Marker Development

SNPs that were closely associated with the AF loci were selected from the GBS data set for the NC493 x CW population for transformation into high-resolution melting (HRM) quantitative PCR markers. PCR primer pairs were designed to span amplicons ranging from 70 to 150 basepairs (bp) flanking the selected SNPs using Primer3 (http://frodo.wi.mit.edu/primer3/). The following criteria were employed for primer pair design: max self-complementarity and max 3’ self-complementarity were set to 4 and 1, respectively; GC content of the primers ranged from 40 to 55%. SNP analysis (Liew et al., 2004) was performed on a LightCycler480 instrument (Roche Diagnostics), and amplifications were performed using the PCR mix and conditions described in Guitton et al. (2011). Outputs were analyzed using the LightCycler480 SW1.5 software. Heterozygous genotypes were identified as having a lower melting temperature in comparison with homozygous genotypes and a shoulder in the melting peaks. HRM markers, which were heterozygous and homozygous for the AF and BF parent, respectively, were screened over the x16.093, x16.109, and x16.111 populations, and association between the HRM genotypes and the presence of the AF trait was assessed using a chi-square test.

### Simple Sequence Repeat Marker Development and Screening

PCR primers for simple sequence repeats (SSRs) from *Rubus* were developed close to the chromosomal regions associated with the loci of interest identified in the NC493 x CW raspberry population, as well as the LG 7 locus identified in blackberry by Castro et al. (2013). SSRs were screened over the C1 blackberry population using a Hitachi ABI3500 Applied Biosystems genetic analyzer (Foster City, CA, USA). Association between the SSR alleles and the presence of the AF trait was determined using a chi-square test.

### RNAseq and Differential Gene Expression Analysis

Total RNA was extracted from axillary buds 5–10 nodes below the apex of ‘Heritage’ and ‘Wakefield,’ which are AF and BF, respectively. The cultivars were grown together in the field under standard conditions at the PFR orchard at Motueka, New Zealand. Bud samples were collected on November 5 (spring/early summer). Tissue was snap-frozen in liquid nitrogen, and total RNA was extracted from three biological replicates of each cultivar, as described in Janssen et al. (2008). The quality and concentration of the RNA samples were assessed using a Fragment Analyzer (Agilent, Santa Clara, CA, USA), and only samples with an RNA Integrity Number higher than 8 were sequenced. Library preparation was completed at the Australian Genome Research Facility using the TruSeq Stranded kit, and subsequent paired-end Illumina^®^ sequencing employed the NovaSeq6000 platform, with the S2 flow cell. An average of ∼19 million, 150-bp paired-end reads was retrieved for each sample (∼6 Gb of data). Read sequences of low-quality ribosomal RNA and adaptors were filtered out using Trimmomatic (Bolger et al., 2014) and SortMeRna (Kopylova et al., 2012). RNAseq reads were aligned to the *R. occidentalis* reference gene models (Vanburen et al., 2018) using Spliced Transcripts Alignment to a Reference (STAR), and differential expression analysis was performed using DESeq2 (Love et al., 2014). All RNAseq data, read statistics, and differentially expressed genes (DEGs) are deposited in NCBI’s Gene Expression Omnibus (Edgar et al., 2002) and are accessible through GEO Series accession number GSE135907 (https://www.ncbi.nlm.nih.gov/geo/query/acc.cgi?acc=GSE135907). Significant DEGs were selected using a threshold of α< -0.005 with an adjusted *p* value of <0.01 and a |log2 fold change | > 1. *Arabidopsis* orthologues were determined by BLAST against the TAIR database.

## Results

### Phenotypic Segregation for AF in Raspberry

The segregating population of 131 F_1_ individuals from the NC493 x CW (AF x BF) cross was assessed for the AF trait over two consecutive years. The observed segregation ratio for AF : BF phenotypes were 55:76 and 66:65 in 2008 and 2009, respectively. Thirty-three phenotypes were inconsistent between years. The subset of the population with consistent AF and BF phenotypes between years was used for GBS analysis. Out of 98 individuals sampled, five did not yield sufficient DNA for GBS and could not be analyzed further. The final set (93 individuals) used for GBS library preparation contained 42 and 51 with AF and BF phenotypes, respectively (**Table S1**).

### GBS of the NC493 x ‘Chilliwack’ Segregating Population

Two lanes of Illumina HiSeq2500 single-end 100 bp reads generated a total amount of 50,515,918,100 bp sequences and 505,159,381 total reads. The removal of adapters and filtering of low-quality reads yielded 46,822,022,484 bp (92% of the total data). The GBS libraries for eight individuals failed to produce any sequencing data. In total, 85 GBS libraries from the progeny and two duplicates of each parent yielded an average number of ∼5.8 million reads per individual that were used for read alignment against the *R. occidentalis* genome assembly ORUS 4115-3 v3.0 (Vanburen et al., 2018) (https://www.rosaceae.org/analysis/268). SNP calling identified 284,146 SNPs in total (**Table 1**). Further filtering of the SNP data was applied to remove loci that had more than 10% missing data and were monomorphic or ambiguous. These filtering criteria yielded a total of 48,002 abxaa and 16,440 aaxab SNPs heterozygous for NC493 and CW, respectively. An additional 5,821 abxab type markers were generated between the parents, resulting in 70,263 SNP markers in total.

**Table 1:** Summary of single-nucleotide polymorphism (SNP) markers obtained by genotyping by sequencing of a mapping population derived from NC493 x ‘Chilliwack’ (CW).

Parents	Total SNPs identified by GBS between parents	Monomorphic SNPs + markers with 10% missing data	abxaa SNPs	abxab SNPs
**NC493**	284,146	230,323	48,002	5,821
**CW**		261,885	16,440	

abxaa type markers are heterozygous in one parent and homozygous in the other parent. abxab type markers are heterozygous for both parents.

### Map Construction

**Figure 1:**
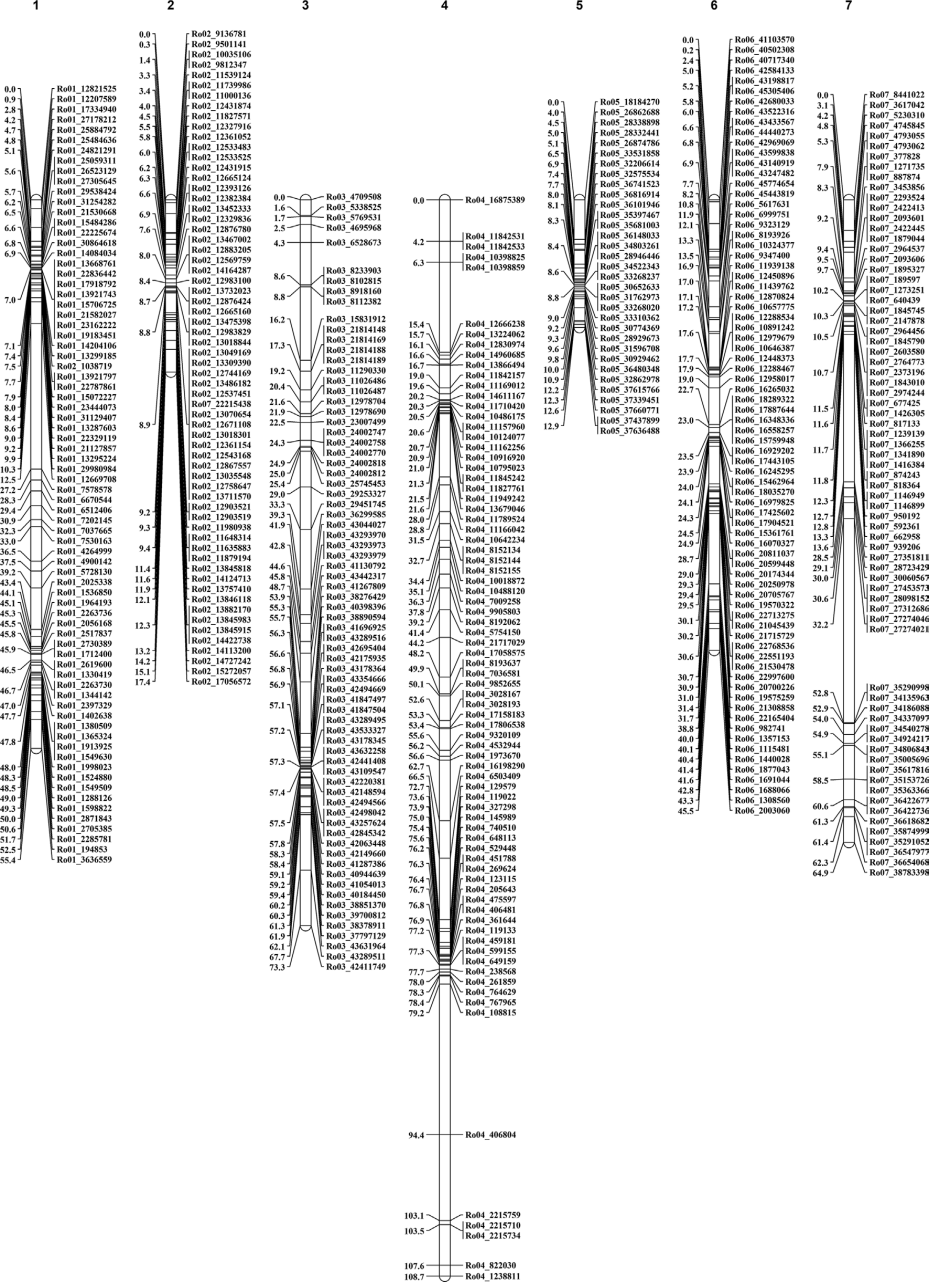
*Rubus idaeus* genetic map for Annual-Fruiting (AF) parent NC493. The vertical bars represent linkage groups, and the lines across the bars represent genotyping by sequencing (GBS) marker position in the map. The scale on the left represents the genetic distance in centiMorgans (cMs). Only markers that segregated according to the Mendelian ratio at *p* < 0.005 were employed for the map construction with Joinmap v5.0^®^ (Van Ooijen and Voorrips, 2001). Each SNP marker was named according its physical position (right-hand side) in the genome.

**Figure 2:**
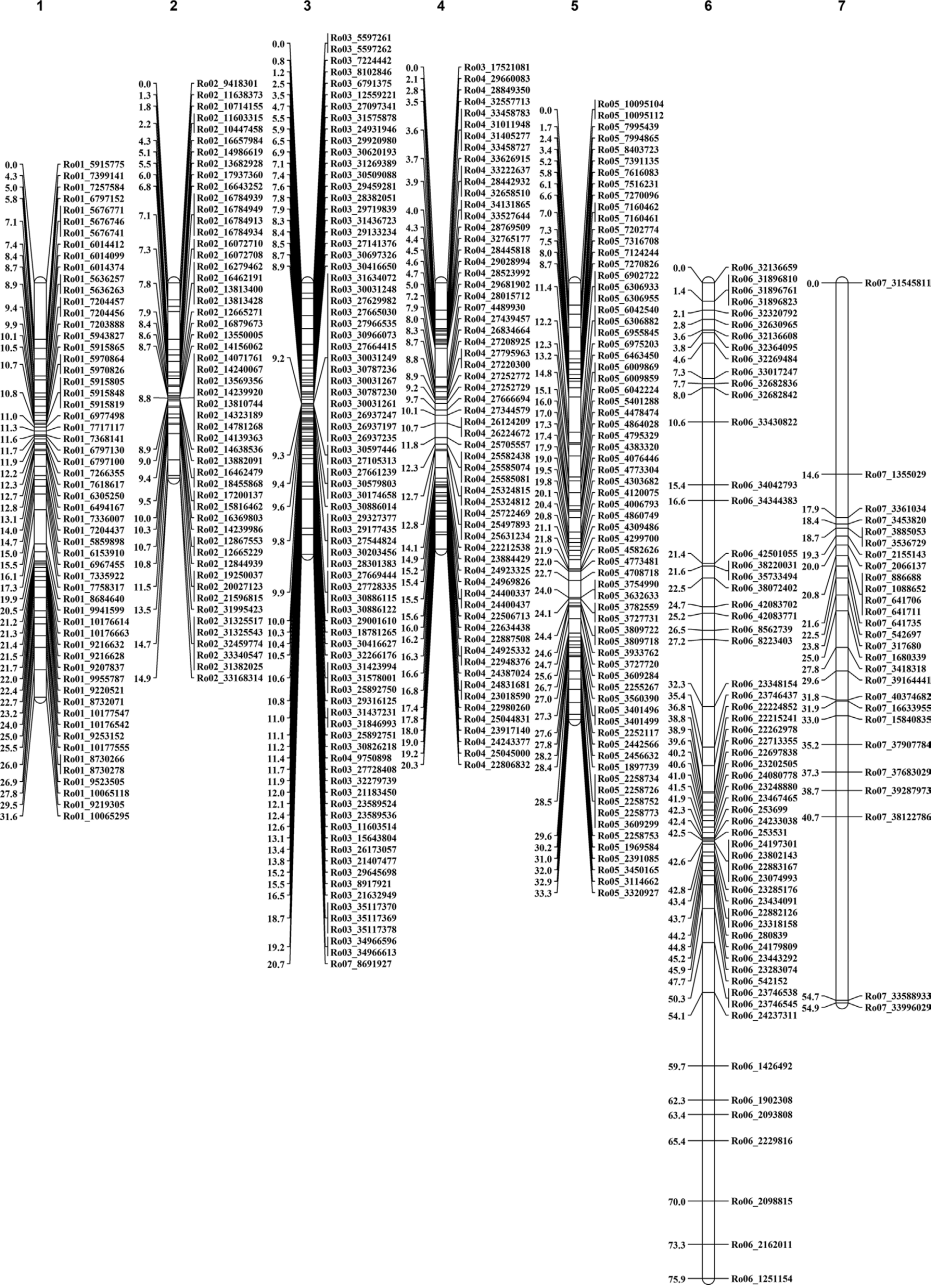
*Rubus idaeus* linkage map for Biennial-Fruiting (BF) parent ‘Chilliwack’. Each linkage group (represented by a vertical bar) was constructed using SNP loci generated by genotyping by sequencing (GBS). The lines across the bars represent marker positions in the map, and the scale on the right represents the genetic distance in centiMorgan (cM). All markers segregated according to the Mendelian ratio at *p* < 0.005, and markers were named according to their physical positions in the genome (right-hand side of each LG). The maps were generated in Joinmap v5.0^®^ (Van Ooijen and Voorrips, 2001).

Linkage maps were constructed for both parents (**Figures 1** and **2**; **Table 2**). The NC493 map comprised 473 markers that spanned the seven LGs, and extended over 378.1 cM, with an average distance of 0.8 cM between markers (**Figure 1**). LG4 had the greatest number of markers (80), LG5 had the fewest number of markers (33), and LG4 was the longest (108.7 cM). The CW parental map was constructed from 419 markers that spanned the seven *Rubus* LGs and covered 251.6 cM, with an average distance of 0.6 cM between markers (**Figure 2**). LG5 had the greatest number of markers (74), and LG7 had fewest markers (27). LG5 was the longest group, with 74 markers covering 75.96 cM.

**Table 2:** Summary of the linkage groups (LG) constructed for NC493 (Annual-Fruiting, AF) and ‘Chilliwack’ (CW, Biennial-Fruiting, BF) parents and the number of markers identified per LG.

Linkage groups	Number of loci mapped in the NC493 map	Number of loci mapped in the CW map
1	75	58
2	64	53
3	73	83
4	80	64
5	33	74
6	76	60
7	72	27
Total markers	473	419

The LGs were constructed by using the SNP markers obtained from a NC493 x ‘Chilliwack’ mapping population.

### Trait Locus Mapping for AF

**Figure 3:**
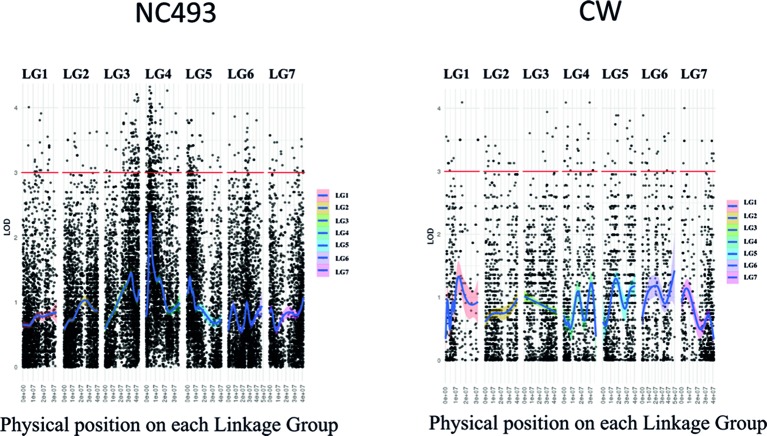
Manhattan plot showing genomic regions associated with Annual-Fruiting (AF) in *Rubus idaeus*. Genotyping by sequencing (GBS)-based single-nucleotide polymorphism (SNP) markers significantly linked to the trait was identified by the chi-square test using markers that were heterozygous in NC493 (AF) and homozygous in ‘Chilliwack’ (Biennial-Fruiting, BF). The x-axis shows LOD scores, and the y-axis indicates the physical position of markers (black points) in the genome divided into seven linkage groups (LGs). The names and the physical position of the markers associated with control of AF are given in **Supplementary Table S2**. CW = ‘Chilliwack’.

The AF trait was not significantly associated with any markers on the saturated CW (BF) linkage map when the AF phenotypes for both years were included in the GBS data set used in map construction. Because the phenotypes were scored qualitatively as presence–absence of AF, they could not be used for interval mapping of QTLs. Hence, the chi-square test was performed on heterozygous GBS markers for each parent to identify markers linked to the AF phenotype (**Table S2**). The analysis of markers heterozygous for NC493 identified two genomic regions, located on LGs 3 and 4, that were significantly associated (LOD > 4) with AF (**Figure 3**). These two new loci were named *RiAF3* and *RiAF4* for *R. idaeus* AF, located on LGs 3 and 4 of NC493, respectively. The GBS markers chr3_41,124,650 and chr4_4,076,592 are those most significantly linked to *RiAF3* and *RiAF4*, respectively. A third locus on LG5 may be present; however, none of the markers were associated with the trait with a LOD score greater than 4. No linkage with phenotype was found for the markers that were informative for CW.

**Figure 4:**
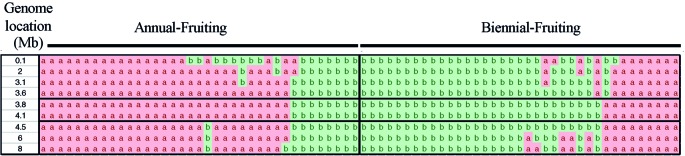
Fine mapping the *RiAF4* locus. A bin map was created by analyzing the genotypes of individuals over small genomic regions located within 0–8 Mb of LG4. Each column represents genotypic data from a single individual, either Annual-Fruiting (AF) or Biennial-Fruiting (BF). The allele of single-nucleotide polymorphisms (SNPs) in the genomic region of LG4 listed in the first column is indicated as a or b. The “a” allele (in pink) and “b” allele (in green) are linked to AF and BF, respectively. Recombination breakpoints are visible as a change in color and narrow the *RiAF4* locus to the region located between 3.6 and 4.1Mb indicated by black horizontal lines.

Examination of the genotypes of 85 individuals over segments of chromosomes 3 and 4 spanning the SNP markers with the most significant LOD scores enabled us to determine the genotypes of the population between 35.1 to 43.7 Mb of chromosome 3 and 0 to 8 Mb of chromosome 4. The mapping analysis using a bin map based on the number of recombinants over a window of 8 Mb indicated that *RiAF4* is located in an interval between 3.50 Mb and 4.38 Mb (**Figure 4**). We were unable to perform similar mapping analysis for *RiAF3* because the order of markers on the linkage map was not colinear with the ORUS 4115-3 v3.0 genome.

### Analysis of *QTLs* in Three Independent Populations of Red Raspberry

**Figure 5:**
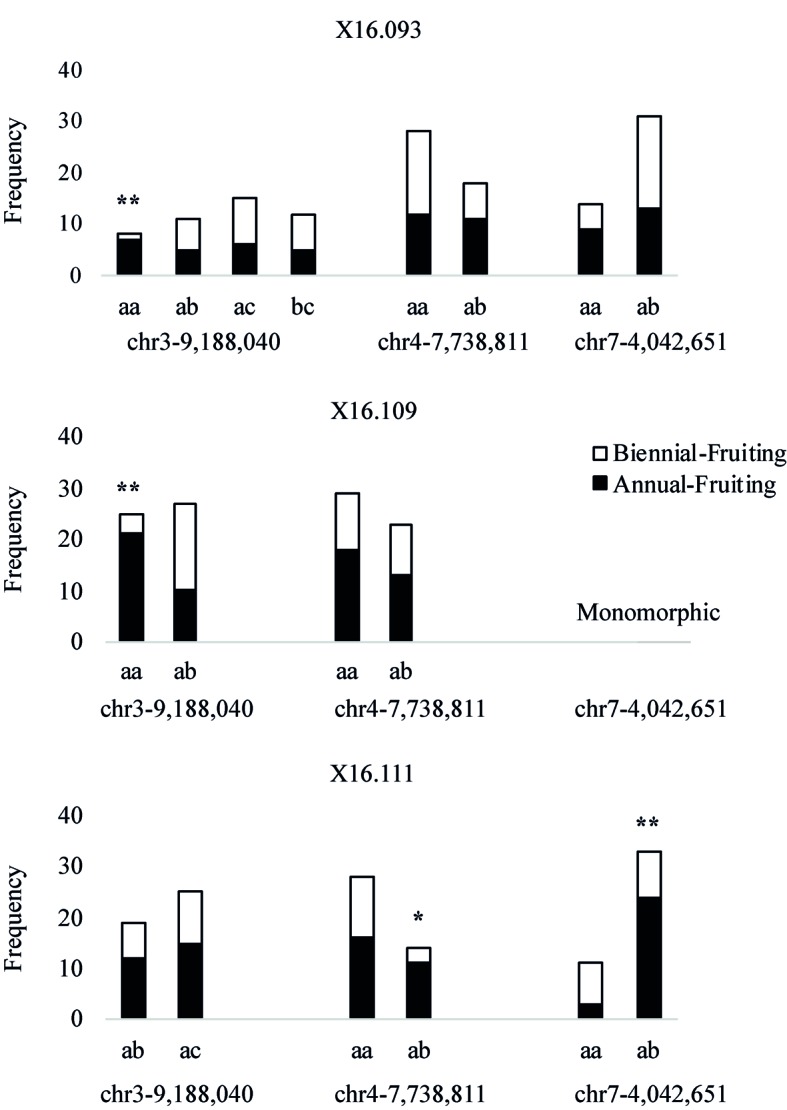
Histograms showing allelic frequencies of SNP-based markers underlying quantitative trait loci in three independent red raspberry populations segregating for the Annual-Fruiting trait. The marker names indicate their chromosome and physical positions (in bp) on the *Rubus occidentalis* genome (Vanburen et al., 2018). The * and ** represent chi-square test significance of 95% and 99% (d.f. = 1), respectively. The marker chr7-4,042,651 was monomorphic in the x16.109 population.

The phenotypes of parents and number of progeny in the three independent populations segregating for the AF trait are shown in **Table 3**. HRM-based markers were developed by designing PCR primer pairs that flanked the most closely linked SNPs on LG3 and LG4 and near the Rubus285a maker on LG7 (**Table S3**). These markers were screened over the three populations (**Figure 5**). In x16.093 and x16.109 populations, the chr3-9,188,040 marker is associated with the AF trait, with chi-square tests significant at 95% and 99% (d.f. = 1), respectively. In population x16.111, ch4-7,738,811 and chr7-4,042,651 markers are associated with the AF trait with chi-square test significance of 95% and 99% (d.f. = 1), respectively.

**Table 3:** Summary of red raspberry (*R. idaeus*) populations segregating for the Annual-Fruiting (AF) trait.

Population	Female parent	Male parent	Total progeny size	AF individuals in progeny	BF individuals in progeny	Chi-square value	P value
x16.093	Z12041-2 (AF)	Z12022-7 (BF)	47	23	24	.01	.950
x16.109	Z12027-13 (AF)	Z12022-7 (BF)	55	32	23	.73	.75
x16.111	Z12027-13 (AF)	Z12011-8 (BF)	49	30	19	1.23	.75

These populations were used for validating RiAF3 and RiAF4 loci. Seedlings were planted in spring of 2017 at the Plant & Food Research site located at Motueka, New Zealand, and were scored for AF in the summer of 2018. BF, Biennial-Fruiting.

### Screening of SSR Markers for *RiAF3*, *RiAF4*, and the LG7 locus in a Blackberry (*R.* subgenus *Rubus*) Population

**Figure 6:**
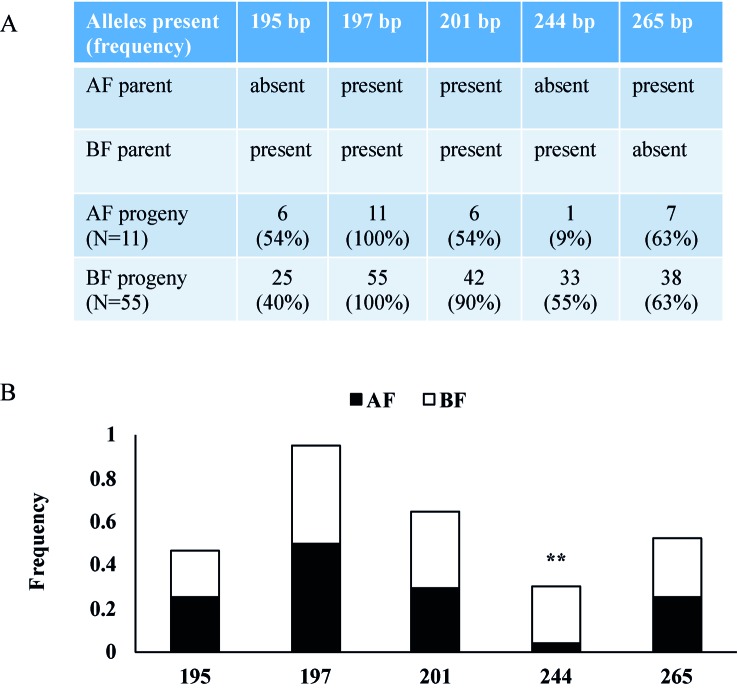
Rubus285a marker segregation in a tetraploid blackberry mapping population segregating for Annual-Fruiting (AF). **(A)** Table showing the presence or absence of alleles in parents and the numbers of AF and Biennial-Fruiting (BF) progeny with each allele and the percentage in parentheses. The AF parent has three alleles with PCR product sizes of 197, 201, and 265 bp. The BF parent has four alleles with sizes of 195, 197, 201, and 244 bp. **(B)** Histogram showing allelic frequencies of the Rubus285a marker in the C1 blackberry population. The 244-bp allele is linked with BF trait with chi-square test significance of 99% (d.f. = 1) indicated by **.

To verify the two newly identified loci, we analyzed markers linked to AF in a tetraploid blackberry population (C1) segregating for the AF trait. In this population, 76 progenies were scored over two consecutive years as 12 AF and 64 BF (**Table S4**). The C1 population was screened with SSR markers from *R.* *idaeus* LGs 3, 4, and 7 (Castro et al., 2013), and only the LG3 marker Rubus285a was associated with AF (**Figure 6**). The data show that the 244-bp allele of Rubus285a from BF parent is linked to the BF phenotype. This allele is not present in the AF parent. No association between marker and phenotype was identified for markers located on LGs 4 and 7.

### Candidate Genes Underlying *RiAF3* and *RiAF4*

The region of the ORUS 4115-3 v3.0 genome spanning from 35.1 to 43.7 Mb on LG3 contains 1,276 predicted gene models, and the region from 0 to 8 Mb on LG4 contains 1,399 gene models (**Table S5**). A number of genes in these intervals are involved with flowering time.

### DEGs Between AF and BF Axillary Buds

To identify genes potentially involved with the transcriptional regulation of AF, we compared gene expression in axillary buds of AF ‘Heritage’ and BF ‘Wakefield’ in late spring. Of the DEG genes with a |log2fold change| > 1, 443 (2.22%) were upregulated, and 363 (1.8%) were downregulated in AF compared with BF. The majority of the DEGs are orthologues of genes identified in other species as being involved in reproduction, flower development, and defence responses against biotic and abiotic stress.

**Figure 7:**
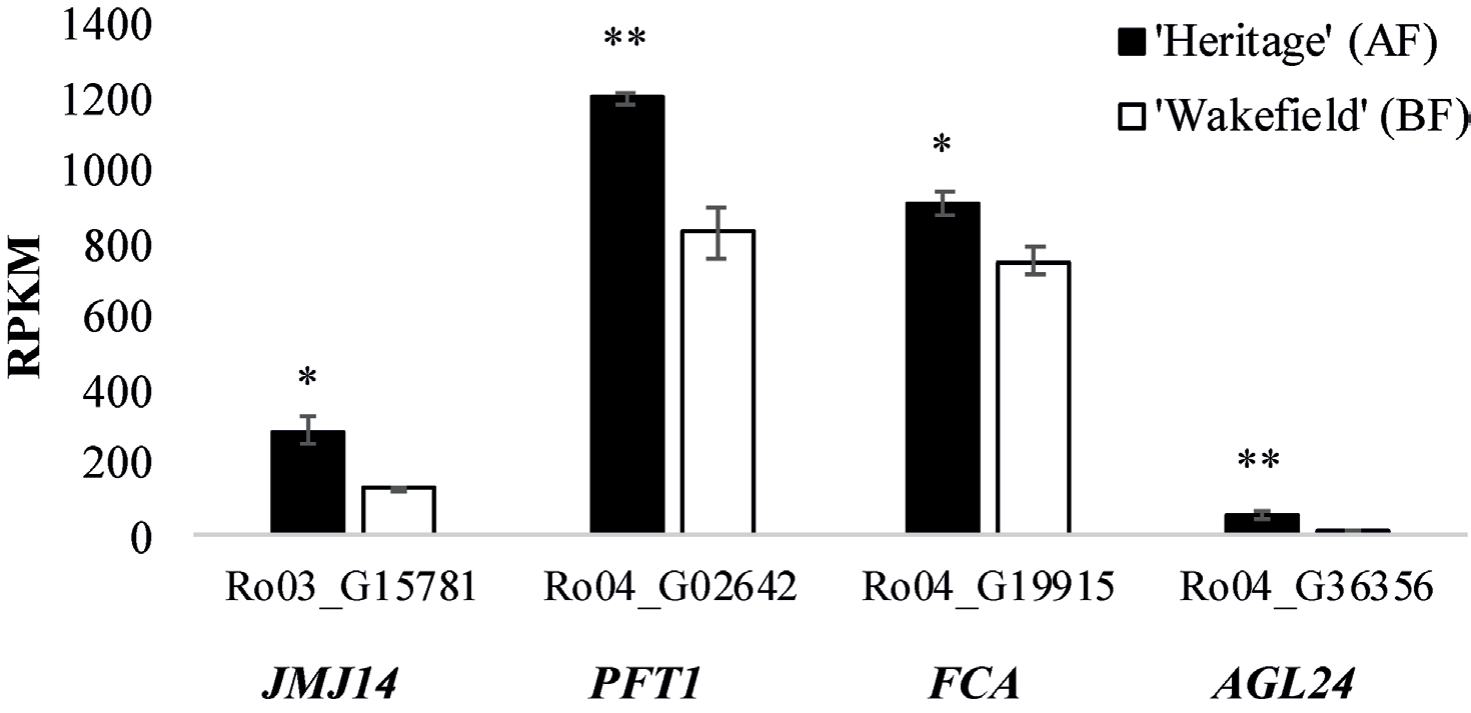
Expression of flowering time genes located in *RiAF3* and *RiAF4* mapping intervals. RNA was collected from axillary buds in late spring from ‘Heritage’ (Annual-Fruiting, AF) and ‘Wakefield’ (Biennial-Flowering, BF). The x-axis shows RPKM (Reads Per Kilobase Million) for Ro03_G15781 (orthologous to *JUMONJI 14*, *JMJ14*), Ro04_G02642 (orthologous to *PHYTOCHROME AND FLOWERING TIME 1*, *PFT1*), Ro04_G19915 (orthologous to *FLOWERING CONTROL LOCUS A*, *FCA*), and Ro04_G36356 (orthologous to *AGAMOUS-LIKE 24*, *AGL24*). The columns represent the means of three biological replicates, and error bars are standard error of the mean. * and **indicate statistically significant differences (*P < 0.05* and *P < 0.01*, respectively) between AF and BF using Student’s *t*-test.

**Table S6** shows the relative expression of all genes within the genomic intervals spanning the *RiAF3* and *RiAF4* loci. The genomic locations and biological functions of some of the most promising differentially expressed candidate genes are listed in **Table 4**. Within the *RiAF3* interval, a gene orthologous to *Arabidopsis* *JUMONJI 14* (*JMJ14*) was expressed 2-fold higher in AF relative to BF. In the *RiAF4* region, *PHYTOCHROME AND FLOWERING TIME 1* (*PFT*1), *FLOWERING LOCUS A* (*FCA*), and *AGAMOUS-LIKE 24* (*AGL24*) genes were all upregulated in AF, with *AGL24* having 5-fold higher transcript levels in AF relative to BF (**Figure 7**).

**Table 4:** Differentially expressed candidate genes that underlie RiAF3 and RiAF4 loci.

*Rubus* gene model	Location in genome (bp)	Log2Foldchange (BF/AF)	*Arabidopsis* hom ologue	Description
Ro04_G19915	8,594,128	-0.28	AT4G16280	Flowering Control Locus A (FCA)
Ro04_G02642	4,485,423	-0.51	AT1G25540	Phytochrome and Flowering Time 1
Ro04_G36356	8,736,093	-1.75	AT4G24540	Agamous-like 24 (AGL24)
Ro03_G05776	35,959,751	-0.001	AT3G12680	Hua1, Enhancer of Ag-4 1
Ro03_G15781	37,388,564	-1.08	AT4G20400	Jumonji 14 (JMJ14)
Ro03_G33037	38,575,123	-0.88	AT1G30330	Auxin Response Factor 6 (ARF6)
Ro03_G13396	39,438,396	1.43	AT1G29390	Cold regulated thylakoid membrane
Ro03_G13391	39,478,286	-0.03	AT4G18130	Phytochrome E
Ro03_G05247	40,252,833	0.50	AT2G42610	Light Sensitive Hypocotyls 10
Ro03_G06544	40,834,857	-0.5	AT1G28330	Dormancy-Associated Protein-like 1
Ro03_G06488	41,189,726	-0.16	AT4G04920	Sensitive to Freezing 6

RNAseq was performed on axillary buds from three biological replicates each of ‘Heritage’ (Annual-Flowering) and ‘Wakefield’ (Biennial-Flowering). The physical location of each R. occidentalis gene model is given in bps (ORUS 4115-3 v3.0, Vanburen et al., 2018). The log2 fold change of ‘Wakefield’ ‘Heritage’ is presented.

## Discussion

We report that AF is a complex genetic trait regulated by at least two loci on *R.* *idaeus* LGs 3 and 4, *RiAF3* and *RiAF4*. These novel loci are syntenic to QTLs identified in strawberry for control of recurrent flowering, suggesting conserved function across the *Rosoideae* subfamily. Markers linked to the newly discovered loci and to a locus previously identified on LG7 (Castro et al., 2013) were tested in independent raspberry and blackberry populations segregating for AF. In addition, we identified DEGs that may be involved in regulating the AF trait in *Rubus*.

### Development of a High-Density Genetic Map of *R. idaeus*

GBS technology has greatly facilitated SNP discovery and genotyping for crop genetics (Crossa et al., 2013; He et al., 2014; İpek et al., 2016; Hackett et al., 2018). We used GBS-based SNP markers to develop a high-density genetic map of red raspberry. This new map is constructed with 70,263 SNP markers and is aligned with the genome assembly of black raspberry. In a previous study, a blackberry genetic map developed with 119 SSR markers in a mapping population segregating for AF (Castro et al., 2013) was employed to identify three markers linked to AF. However, one marker was 71cM from AF and, hence, unlinked. Furthermore, none of their LG7 markers mapped on the black raspberry genetic map of Bushakra et al. (2012) and the blackberry LG7 was assigned by default. It is possible that one of the other six LGs could have split and been mistakenly designated LG7 by Castro et al. (2013).

### AF Is a Complex Genetic Trait

Castro et al. (2013) proposed that AF in blackberry was controlled by a single locus located on LG7. We were unable to map the AF phenotype as a single locus in the NC493 x CW population. A chi-square test of the thousands of markers detected by GBS identified two novel loci on LGs 3 and 4 for control of AF in red raspberry. The previous single locus hypothesis was largely based on the 3:1 (BF : AF) phenotypic ratio observed in several populations but is unsupported by molecular marker data (Oberle and Moore, 1952; Keep, 1961). On the basis of the phenotypic data collected from intercrossing or selfing AF individuals, some studies have suggested a complex genetic basis for the trait (Lewis, 1939; Waldo and Darrow, 1941; Haskell, 1960; Fejer and Spangelo, 1974; Fejer, 1977). Various studies concluded that AF is controlled by a number of minor genes with predominant evidence that AF is a complex genetic trait controlled by loci on three LGs (*RiAF3*, *RiAF4*, and LG7). Recently, linkage analysis of ‘Glen Moy’ x ‘Latham’ raspberry population identified flower development QTLs on LGs 3, 5, and 7 (Hackett et al., 2018). These QTLs harbored genes involved in regulating flowering time. For example, FKF1, a regulator of CO expression, was mapped to LG7. Similarly, FT, EFL7 (a regulator of FLC levels), and COL9 (a regulator of CO levels) were mapped to LG3.

In this study, we found that LGs 3, 4, and 7 HRM markers were linked to the AF trait in red raspberry (**Figure 5**). An LG3 SSR marker (Rubus285a) was linked to the BF trait in blackberry (**Figure 6**). We were unable to verify all three loci in all of the populations, which could be due to one or more loci being fixed in a homozygous state in the parents, hence preventing detection of polymorphic markers linked to AF.

Our GBS data indicated that *RiAF3* is located at the bottom of chromosome 3 (**Figure 3**). However, the HRM marker that was developed from the most closely linked SNP is located on the upper arm of chromosome 3 (chr3-9,188,040). This discrepancy is likely due to errors in the assembly, and it is possible that the HRM marker position is incorrect. The populations used for the QTL analysis were relatively small, which would reduce the possibility of detecting several loci. This analysis should be repeated in one or more large populations to more precisely identify the genomic intervals linked to AF.

### *RiAF3* and *RiAF4* Are Syntenic With Two Loci for Control of Recurrent Flowering in Strawberry

To extend the fruiting season, many international strawberry breeding programs are focusing on developing early, late, and perpetual (continuous) flowering cultivars. Comparative genomic studies between strawberry and raspberry have revealed a high degree of synteny between the genomes of two species (Bushakra et al., 2012; Jibran et al., 2018). Results from molecular studies also support a common molecular mechanism for control of flowering time among different Rosaceae species (Gaston et al., 2013; Honjo et al., 2016; Perrotte et al., 2016; Samad et al., 2017). Floral repressors, such as *Perpetual Flowering 1* and *Terminal Flower 1*, control perpetual flowering habits in strawberry. Seasonal flowering (SF) strawberry plants produce flowers only in autumn, whereas perpetual flowering (PF) plants flower over an extended time. The strawberry PF habit is under the control of a major QTL named *FaPFRU*, located on the lower arm of LG4 called LG4b-F (Gaston et al., 2013). This mapped region contains a floral activator gene orthologous to *Flowering Time* (*FT*) (Yoo et al., 2005). Perrotte et al. (2016) investigated the effect of *FaPFRU* on PF habit in 28 strawberry genotypes and found that the locus was linked to PF when analysis was carried out in both PF and SF genotypes. However, the analysis involving only PF genotypes did not detect linkage of *FaPFRU* with the PF trait; instead, a QTL located on LG3c-F (LG3c), associated with a late PF-intense phase, was identified. Hence, the authors concluded that PF in strawberry is regulated by multiple loci. It was postulated that *FaPFRU* regulates the switch between PF and SF, whereas the LG3c locus controls the intensity of flowering. Furthermore, Albani et al. (2004) suggested that the PF trait in woodland strawberry (*Fragaria vesca* L.) and cultivated strawberry are regulated by different genetic components. In woodland strawberry, two early flowering QTLs were identified on the upper and lower arm of LG4 (Samad et al., 2017).

The two novel loci *RiAF3* and *RiAF4* identified from the NC493 x CW population colocalize with the previously identified QTLs related to PF in strawberry. *FaPFRU* is located on the lower arm of LG4, whereas *RiAF4* locus is located at the upper arm of LG4. The difference in the genome positions between species might well be because parts of LG4 are inverted in raspberry compared to strawberry (Vanburen et al., 2018).

### Candidate Genes Underlying *RiAF3* and *RiAF4*

*JMJ14* is the best candidate for *RiAF3*. JMJ14 is a histone H3 lysine 4 (H3K4) demethylase, and H3K4 methylation is linked to transcription of key flowering time genes (Lu et al., 2010; Lu et al., 2011; Cui et al., 2016). The *Arabidopsis* *jmj14-1* mutant flowers early under short day conditions and has elevated levels of *LFY*, *FT*, and *AP1* transcripts (Jeong et al., 2009). Previous studies have indicated that demethylases are involved with the regulation of flowering time. Yang et al. (2012) found that *Arabidopsis* plants overexpressing *JMJ15*, a member of the *H3K4 demethylase* *JARID1* family, had accelerated flowering time. The early flowering phenotypes of the overexpression lines were associated with an increased *FT* expression and a decrease in H3K4me3 at the *FLC* locus that cause FLC repression. It has also been shown that *JMJ14* is required for gene silencing (Searle et al., 2010). Similarly, Zheng et al. (2019) found that *JMJ13* is a floral repressor that regulates *Arabidopsis* flowering timing in a temperature- and light-dependent manner.

The genomic region underlying the *RiAF4* locus contains three key genes that promote flowering, *PFT1* (Ro04_G02642), *FCA* (Ro04_G19915), and *AGL24* (Ro04_G36356) (Simpson et al., 2003; Turck et al., 2008; Michaels et al., 2003; Torti and Fornara, 2012). PFT1 is a nuclear protein that integrates various environmental cues into plant flowering pathway both in a *CO*-dependent and -independent manner (Liu et al., 2017). *PFT1* regulates flowering by increasing the transcript abundance of *CO* and *FT* (Iñigo et al., 2012). *FCA* is an RNA binding protein that downregulates expression of the floral repressor FCA by methylating central parts of the FLC gene (Liu et al., 2007). Reduced *FCA* function results in late flowering due to increased FLC activity, whereas overexpression of *FCA* causes early flowering, both in long and short day photoperiods (Liu et al., 2007). *AGL24* is a MADS-box transcription factor that regulates flower timing by inducing expression of the floral integrator SOC1 (Liu et al., 2008). *AGL24* loss-of-function mutants and plants with reduced *AGL24* transcript levels showed delayed flowering phenotypes, whereas overexpression of AGL24 resulted in early flowering phenotypes (Michaels et al., 2003; Yu et al., 2002). It was suggested that AGL24 controls flower timing in a dosage-dependent manner (Yu et al., 2002). Our finding that the raspberry orthologues of *PFT1, FCA*, and *AGL24* are upregulated in AF buds relative to BF makes each of them promising candidate genes in the control of AF.

## Conclusion

We have presented the first evidence of loci linked to control of AF in *R. idaeus*. Our results suggest that two major loci *RiAF3* and *RiAF4* and a region located on the upper arm of LG7 control AF in *Rubus*. Additionally, we identified putative flowering time genes as candidates for functional validation. The genetic loci identified will be of value for marker-assisted selection of AF raspberries and blackberries following further validation in breeding germplasm.

## Data Availability Statement

All datasets for this study are included in the article/**Supplementary Material**.

## Author Contributions

RJ, substantial contributions to the conception or design of the work; or the acquisition, analysis or interpretation of data for the work. JS, contribution to labwork and fieldwork. GH, acquisition, analysis or interpretation of data for the work. AM, acquisition, analysis or interpretation of data for the work. MM, contribution to labwork and fieldwork. HD, contribution to data analysis. JT, contribution to data analysis. KD, comments to the manuscript. DC, substantial contributions to the conception or design of the work; or the acquisition, analysis or interpretation of data for the work. TF, substantial contributions to the conception or design of the work; or the acquisition, analysis or interpretation of data for the work.

## Conflict of Interest

Author RJ is employed by The New Zealand Institute for Plant & Food Research Limited. The remaining authors declare that the research was conducted in the absence of any commercial or financial relationships that could be construed as a potential conflict of interest.
